# A fast structural multiple alignment method for long RNA sequences

**DOI:** 10.1186/1471-2105-9-33

**Published:** 2008-01-23

**Authors:** Yasuo Tabei, Hisanori Kiryu, Taishin Kin, Kiyoshi Asai

**Affiliations:** 1 Graduate School of Frontier Science, University of Tokyo, CB04 Kiban-tou 5-1-5 Kashiwanoha, Kashiwa, Chiba, 277-8561, Japan; 2 Computational Biology Research Center (CBRC), National Institute of Advanced Industrial Science and Technology (AIST), 2-42 Aomi, Koto-ku, Tokyo, 135-0064, Japan

## Abstract

**Background:**

Aligning multiple RNA sequences is essential for analyzing non-coding RNAs. Although many alignment methods for non-coding RNAs, including Sankoff's algorithm for strict structural alignments, have been proposed, they are either inaccurate or computationally too expensive. Faster methods with reasonable accuracies are required for genome-scale analyses.

**Results:**

We propose a fast algorithm for multiple structural alignments of RNA sequences that is an extension of our pairwise structural alignment method (implemented in SCARNA). The accuracies of the implemented software, MXSCARNA, are at least as favorable as those of state-of-art algorithms that are computationally much more expensive in time and memory.

**Conclusion:**

The proposed method for structural alignment of multiple RNA sequences is fast enough for large-scale analyses with accuracies at least comparable to those of existing algorithms. The source code of MXSCARNA and its web server are available at .

## Background

Non-coding RNAs (ncRNAs) are transcribed RNA molecules that do not encode proteins. Their functions often depend on their 3D-structures rather than their primary sequences. The secondary structures of RNA sequences can be identified by various methods, including minimization of the free energy [1-3]. However, it is not always possible to obtain the accurate secondary structures. More reliable predictions of the secondary structures are possible if we have a set of RNA sequences with a common secondary structure. For consensus structure prediction, RNAalifold [4], Pfold [5], and McCaskill-MEA [6] are applicable only to sets of aligned RNA sequences. Multiple alignment tools that consider only sequence similarities, e.g. ClustalW [7], Dialign [8], and T-Coffee [9], however, have limited accuracy for RNA sequences with low similarity.

Simultaneous prediction of the common secondary structure and optimal alignment of RNA sequences is computationally quite expensive, even if pseudo-knotted structures are excluded. For example, the strict algorithm of Sankoff [10] requires *O*(*L*^3*N*^) in time and *O*(*L*^2*N*^) in memory for *N *sequences of length *L*. Its faster variants that restrict the distances of the base pairs in the primary sequences are proposed for pairwise alignments [11-14].

Although structural alignment of multiple RNA sequences with reasonable computational cost is difficult, several algorithms have been proposed. Hofacker et al. proposed a method for progressive multiple alignments by direct comparison of the base-pairing probability matrices [12], implemented in PMmulti which was recently reimplemented in FoldalignM [15] and Locarna [16] by Torarinsson et al. and Will et al., respectively. In Stemloc, Holmes et al. incorporated a constraint approach that limits the range of structures and alignments to be considered by pre-processing the sequences [13,14]. Siebert et al. proposed an approach distantly related to Sankoff's algorithm and implemented it in MARNA [17] that uses the structural information for pairwise alignments before combining them into multiple alignments with T-Coffee [9]. Dalli et al. developed a new scoring approach, StrAl, that takes into account sequence similarities as well as base-pairing probabilities [18]. Xu et al. proposed a new sampling based algorithm that finds the common structure between input sequences by probabilistically sampling aligned stems based on stem conservation calculated from intrasequence base pairing probabilities and intersequence base alignment probabilities, which was implemented in RNASampler [19]. Bauer et al. developed a graph based representation which modeled sequence-structured alignment as an integer linear program (ILP), and implemented it in RNAlara [20]. Kiryu et al. proposed a variant of Sankoff's algorithm with marked reduction of computation, which was implemented in Murlet [21]. All of these methods, however, are still too slow to apply to the RNA sequences longer than 1000 bases. Seibel et al. developed an alignment tool with an editor, which uses the secondary structure information of individual sequences to align multiple RNA sequences with low time complexities (4SALE) [22]. In order to extract the common secondary structure, it is also possible to find the structural motifs without aligning the whole sequences. For structural motif finding, Yao et al. proposed an algorithm based on covariance models (CMfinder) [23], and Hamada et al. proposed a graph mining approach (RNAmine) [24].

Here we propose a method, implemented in MXSCARNA, for fast multiple alignments of RNA sequences. This method extends our previous work in pairwise alignments (SCARNA) [25] to progressive multiple alignments with improved score functions, and simultaneously construct multiple alignments and the associated common secondary structures. The pairwise alignment in this progressive alignment is an heuristic algorithm that separately aligns 5' parts and 3' parts of the stems with rough consistency considerations.

In benchmark experiments, our method was at least as accurate as currently available state-of-art multiple alignment methods, but unlike those methods, the computations were fast enough for large-scale analyses, though the accuracies for the alignments of long sequences have not yet been confirmed.

## Results and Discussion

### Algorithm

#### Overview of the algorithm

The proposed method, implemented in MXSCARNA, progressively aligns multiple RNA sequences, in an extension of the pairwise structural alignment algorithm (implemented in SCARNA) of our previous work [25].

First the guide tree for the progressive alignment is built by Unweighted Pair Group Method with Arithmetic Mean (UPGMA) [26] by using the pairwise similarities of the RNA sequences. Second the base-pairing probability matrices are calculated for all the RNA sequences by McCaskill's algorithm [27]. Those base-pairing probabilities are used for extracting the potential stems and for the matching scores in the Dynamic Programming (DP) of the alignments. Third the RNA sequences are progressively aligned along the guide tree using SCARNA's pairwise alignment algorithm with improved score functions introduced in this paper.

At the first stage of the progressive alignment, which corresponds to the bottom level of the guide tree, the pairs of RNA sequences are aligned by engineered DP algorithm of SCARNA's pairwise alignment. The pairwise alignment is very fast because the potential stems extracted from the base-pairing probability matrices are decomposed into 5' part and 3' part and those two parts are independently aligned. In each upper-level step of the progressive alignment according to the guide tree, potential stems for groups of RNA sequences are extracted from the averaged base-pairing probability matrices.

The DP algorithm of the pairwise alignment uses the approximated posterior probabilities as score functions. The approximation uses the product of the pairwise posterior probabilities of Maximum Expected Accuracy (MEA) alignments and the base-pairing probabilities of the sequences. MEA alignment maximizes the expected number of positions where the two nucleotides are correctly aligned. To yield robust alignments, the pairwise posterior probabilities of MEA alignments are modified by the probability consistency transformation.

#### Definitions

##### Definition 1: Stem candidate

Given a base-pairing probability matrix for an RNA sequence and a threshold *τ *(0 <*τ *< 1), *stem candidate *is a set of continuous base pairs of which the base-pairing probabilities are greater than *τ*.

##### Definition 2: Stem fragment

Given a base-pairing probability matrix for an RNA sequence, a threshold *τ *(0 <*τ *< 1), and an integer *W*, *stem fragment *is a set of continuous base pairs of length *W*, of which the base-pairing probabilities are greater than *τ*.

**Figure 1:**
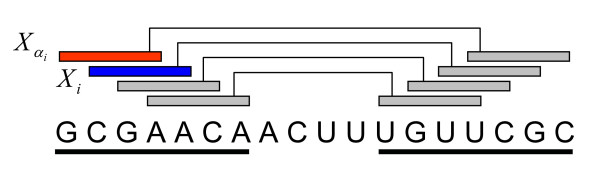
**Stem candidates, stem fragments and stem components**. A stem candidate (a pair of underlined positions) comprises four overlapping stem fragments. A fragment consists of a 5' (left) component and a 3' (right) component. *X*_*i *_(blue box) and $X_{\alpha_{i}}$ (red boxe) are 1-continuous stem components.

A stem candidate longer than *W *is represented by a set of overlapping stem fragments of fixed-length *W *(Figure 1). Smaller values in *W *or *τ *increase the sensitivity of the predictions of the stems and decrease the specificity of them. *W *and *τ *are set to 2 and 0.01 respectively in all the computational experiments in this paper. For each stem fragment, the *5' stem component *and the *3' stem component*, which are representatives of the 5' and 3' portions of the stem fragment, respectively, are defined as follows.

##### Definition 3: Stem component

For each stem fragment, a *stem component X*_*a*_, either a 5' stem component or a 3' stem component, is an object that has the following properties:

• *p*(*X*_*a*_): position, the position of the leftmost base of the 5' or 3' part of the stem fragment.

• *s*(*X*_*a*_): sequence, the nucleotide sequence of the 5' or 3' part of the stem fragment.

• *c*(*X*_*a*_): partner component, the complementary (3' or 5') stem component.

• *d*(*X*_*a*_): loop distance, the distance to the complementary (3' or 5') stem component.

A stem fragment is written as [*X*_*a*_, *X*_*a'*_] by using the mutually complementary stem components, 5' stem component *X*_*a *_and 3' stem component *X*_*a'*_, which represent the 5' and 3' parts of the stem fragment. *X*_*a *_and *X*_*a' *_satisfy

*X*_*a *_= *c*(*X*_*a'*_) and *X*_*a' *_= *c*(*X*_*a*_).

The loop distance *d*(*X*_*a*_) can be written as

*d*(*X*_*a*_) = *p*(*c*(*X*_*a*_)) - *p*(*X*_*a*_) - W.

##### Definition 4: stem component sequence (SCS)

A *stem component sequence *(SCS) is a sorted sequence of all the stem components of an RNA sequence, in order of their positions and, if the positions are the same, according to their loop distances.

For *i *<*j*, a SCS ***X ***= *X*_1_*X*_2 _... *X*_*m *_satisfies

*p*(*X*_*i*_) <*p*(*X*_*j*_) or *p*(*X*_*i*_) = *p*(*X*_*j*_) &*d*(*X*_*i*_) <*d*(*X*_*j*_).

##### Definition 5: relations of stem fragments without an overlap

Two stem fragments, [*X*_*a*_, *X*_*a'*_] and [*X*_*b*_, *X*_*b'*_] of an RNA sequence are, *parallel *if and only if

*p*(*X*_*a*_) <*p*(*X*_*a'*_) <*p*(*X*_*b*_) <*p*(*X*_*b'*_) or *p*(*X*_*b*_) <*p*(*X*_*b'*_) <*p*(*X*_*a*_) <*p*(*X*_*a'*_),

*nested *if and only if *p*(*X*_*a*_) <*p*(*X*_*b*_) <*p*(*X*_*b'*_) <*p*(*X*_*a'*_) or *p*(*X*_*b*_) <*p*(*X*_*a*_) <*p*(*X*_*a'*_) <*p*(*X*_*b'*_),

*pseudo-knotted *if and only if *p*(*X*_*a*_) <*p*(*X*_*b*_) <*p*(*X*_*a'*_) <*p*(*X*_*b'*_) or *p*(*X*_*b*_) <*p*(*X*_*a*_) <*p*(*X*_*b'*_) <*p*(*X*_*a'*_).

##### Definition 6: relations of overlapping stem fragments

Two stem fragments, [*X*_*a*_, *X*_*a'*_] and [*X*_*b*_, *X*_*b'*_] of an RNA sequence are, *r-continuous *if and only if

*r *= *p*(*X*_*b*_) - *p*(*X*_*a*_) = *p*(*X*_*a'*_) - *p*(*X*_*b'*_),

*ill-continuous *if and only if *X*_*a *_overlaps *X*_*b *_and *X*_*a' *_overlaps *X*_*b' *_and

*p*(*X*_*b*_) - *p*(*X*_*a*_) ≠ *p*(*X*_*a'*_) - *p*(*X*_*b'*_),

*contradictory *if and only if only one side, either 5' part or 3' part, of the stem fragments overlap.

The three possible relationships between stem fragments without an overlap: parallel, nested, and pseudo-knotted, may exist in the same secondary structure of an RNA sequence. However, among the three possible relationships between overlapping stem fragments, only r-continuous stem fragments may coexist in the same secondary structure of an RNA sequence. *1-continuous*, a special case of r-continuous, means that the two stem fragments are adjacent in the RNA sequence and a part of a stem candidate with a length of *W *+ 1 (Figure 1). As described later, two overlapping stem components in the alignment are controlled to belong to two r-continuous stem fragments in DP.

#### Building stem component sequences

In a base-pairing probability matrix, which is calculated by McCaskill's algorithm [27], a potential stem is located in two symmetry locations as continuous counterdiagonal positions which have high base-pairing probabilities. Therefore, the stem components for each RNA sequence defined in previous section are extracted by scanning counterdiagonal windows of length *W *in the base-pairing probability matrix and selecting the windows whose elements are greater than *τ*. Smaller value in *W *or *τ *increase the sensitivity of the predictions of the stems and decrease the specificity of them. *W *and *τ *are set to 2 and 0.01 respectively in all the computational experiments in this paper.

The stem components are sorted in order of their positions and loop distances to construct a stem component sequence (SCS).

For each group alignment in the progressive alignment, the average of the base-pairing probability matrices is calculated directly according to the alignment of the group of RNA sequences. The stem components for the group are extracted from the averaged matrix, and the SCS is constructed by sorting the stem components.

#### Alignment of stem component sequences

Before the pairwise alignments or group alignments, RNA sequences or groups of RNA sequences are represented by their stem component sequences (SCSs). Those two SCSs are aligned by SCARNA's pairwise DP algorithm in each stage of the progressive alignment. The alignment of the two SCSs uses two DP matrices, *M*(*i*, *j*) and *G*(*i*, *j*). For two SCSs, {*X*_*i*_}(*i *= 1, ... |***X***|) and {*Y*_*j*_}(*j *= 1, ... |***Y***|), *M*(*i*, *j*) is the best score of the alignment of the pair *X*_*i *_and *Y*_*j*_, given that *X*_*i *_matches *Y*_*j*_, and *G*(*i*, *j*) is the best score given that *X*_*i *_mismatches *Y*_*j*_. The recursions for *M*(*i*, *j*) and *G*(*i*, *j*) are written as:

(1) $$M(i,j)=\max\{\begin{array}{l} M(\alpha_{i},\beta_{j})+\delta_{s}(i,j)\\ M(p_{i},q_{j})+s(i,j)\\ G(p_{i},q_{j})+s(i,j) \end{array}$$

(2) $$G(i,j)=\max\{\begin{array}{l} M(i-1,j)\\ M(i,j-1)\\ G(i-1,j)\\ G(i,j-1) \end{array}$$

with the initial conditions; *M*(0, 0) = 0, *M*(·, 0) = *M*(0, ·) = *G*(0, 0) = *G*(·, 0) = *G*(0, ·) = -∞.

**Figure 2:**
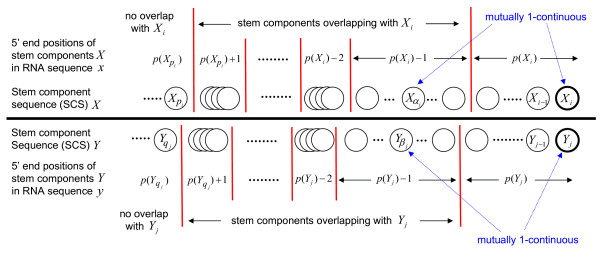
**An example of relations of indices of stem components in SCS alignment**. *α*_*i*_/*β*_*j *_and *p*_*i*_/*q*_*j *_in equation (1) are the indices (smaller than *i*/*j*) of stem components of *X*/*Y*. The red lines separate the stem components into groups which have the same positions in RNA sequences. When *X*_*i *_and *Y*_*j *_match in DP of SCS alignment, the stem components of the adjacent previous match must be either non-overlapping or 1-continuous with *X*_*i*_/*Y*_*j*_. $X_{\alpha_{i}}/Y_{\beta_{j}}$ are 1-continuous with *X*_*i*_/*Y*_*j*_. $X_{p_{i}}/Y_{q_{j}}$ are the nearest stem components that do not overlap with *X*_*i*_/*Y*_*j*_.

The first term of equation (1) controls the *1-continuous *case where the continuous matches of two overlapping stem components form a match of the corresponding stem longer than *W*. *α*_*i*_/*β*_*j *_are the indices (smaller than *i*/*j*) of the components that are 1-continuous with *X*_*i*_/*Y*_*j*_. The positions of $X_{\alpha_{i}}/Y_{\beta_{j}}$ are adjacent to *X*_*i*_/*Y*_*j *_in the nucleotide sequences (Figures 1 and 2), i.e.

$$\begin{array}{ll} p(X_{i})=p(X_{\alpha_{i}})+1, & p(c(X_{i}))=p(c(X_{\alpha_{i}}))-1,\\ p(Y_{j})=p(Y_{\beta_{j}})+1, & p(c(Y_{j}))=p(c(Y_{\beta_{j}}))-1 \end{array}$$

*δ*_*s*_(*i*, *j*) corresponds to the incremental score for the match of the overlapping stem components, which is discussed in the next section.

The second and third terms of equation (1) keep the stem components in the adjacent DP match from overlapping in the nucleotide sequences. *p*_*i*_/*q*_*j *_are the indices (smaller than *i*/*j*) of the nearest components that do not overlap with *X*_*i*_/*Y*_*j *_(Figure 2). *s*(*i*, *j*) is a match score for *X*_*i *_and *Y*_*j*_, which is discussed in the next section.

Equation (2) refers only adjacent positions in DP matrix because overlaps of *X*_*i *_and *Y*_*j *_with the other stem components are permitted. Because the 5' stem components and the 3' stem components are handled independently, there is no term for bifurcation in secondary structures in equations (1) and (2).

The traceback pointer keeps the triplets, indices of ***X***, ***Y***, and the selection of *M *or *G*, in the recursion (1) and (2). The first term of the triplet, the index of ***X***, can be either *α*_*i*_, *p*_*i*_, *i*, or *i *- 1, and the second term of the triplet, the index of ***Y***, can be either *β*_*j*_, *q*_*j*_, *j*, or *j *- 1. In the traceback of the DP, *M*(*i*, *j*) and *G*(*i*, *j*) are used jointly to obtain the optimal path and to select *M *or *G*, which gives the maximum score of the alignment. The alignments of SCSs are constructed by selecting the stem components that appear in the path with the selected *M*. All of the mismatched stem components are excluded from the alignment. The algorithm makes the adjacent DP matches of stem components either not overlapping in the nucleotide sequences or consistently overlapping (*1-continuous*) as a match of the stems longer than *W*. Pairwise alignment of the SCSs requires only *O*(|***X***||***Y***|) in time and in memory. That computational complexities are evaluated as (*L*^2^) for two RNA sequences of length *L *because the number of the stem components is regarded as a linear function of the length of the nucleotide sequence [25].

The pairwise alignment of SCSs allows some inconsistent matches by ignoring strict treatments of the complementary components. For two stem fragments, [*X*_*a*_, *X*_*a'*_] and [*Y*_*b*_, *Y*_*b'*_], if *X*_*a *_matches *Y*_*b *_in the SCS alignment, *X*_*a' *_should match *Y*_*b'*_. Let us define such a match as left-right consistent. Because 5' stem components and 3' stem components are aligned independently, left-right consistency is not guaranteed in general. Any match which is not left-right consistent is removed as a post process. If any two of the stem components of a same SCS appear in the SCS alignment and their complementary components overlap (i.e. contradictory in Definition 6), those complementary components do not appear together in the alignment because the alignment of complementary components are controlled to be either nonoverlapping or r-continuous. Therefore, the post process also guarantees that no pair of contradictory stem fragments appears in the alignment [25].

#### The score function using the MEA alignment

In our previous work [25], a function of the RIBOSUM [28] score, loop distance, base-pairing probabilities, and the stacking energy were used as the score *s*(*i*, *j*) in recursion (1). In MXSCARNA, the score function is replaced by an approximated posterior probability according to the principle of Maximum Expected Accuracy (MEA). Recent studies have shown that the accuracy of the resulting sequence alignment and secondary structure predictions is better than that of predictions made by the conventional maximum likelihood estimation (MLE) algorithms [21,29-32].

In the following, for nucleotide sequences ***x ***and ***y***, *x*_*i *_~ *y*_*j *_means that *x*_*i *_∈ ***x ***and *y*_*i *_∈ ***y ***are aligned on the same column in the alignment, and *x*_*i *_◇ *x*_*j *_means that *x*_*i*_, *x*_*j *_∈ ***x ***form a base pair. For two RNA sequences, ***x***, ***y ***and *k*, *l *∈ {1, ···, |***x***|}, *m*, *n *∈ {1, ···, |***y***|}, let *P*(*x*_*k *_~ *y*_*m*_, *x*_*l *_~ *y*_*n*_, *x*_*k *_◇ *x*_*l*_, *y*_*m *_◇ *y*_*n*_|***x***, ***y***) be the posterior probability, i.e. the sum of the probabilities that two positions of the sequences, *x*_*k *_and *y*_*m*_, *x*_*l *_and *y*_*n*_, are aligned in the alignment, and that two pairs of the nucleotides, *x*_*k *_and *x*_*l*_, *y*_*m *_and *y*_*n*_, form base pairs in the secondary structures as well; this is computed by the inside-outside algorithm of the pair Stochastic Context Free Grammar (pair SCFG) [5] for structural pairwise alignments of RNA sequences. We wanted to use posterior probability as the score function *s*(*i*, *j*), but the computational costs, *O*(*L*^6^) in time and *O*(*L*^4^) in memory for sequences of length *L*, are impractical. We instead used the following approximated posterior probability introduced by Kiryu et al. [21].

$$\begin{array}{ll} & \hat{P}(x_{k}\sim y_{m},x_{l}\sim y_{n},x_{k}⋄x_{l},y_{m}⋄y_{n}|x,y)\\ = & \tilde{P}(x_{k}\sim y_{m}|x,y)\tilde{P}(x_{l}\sim y_{n}|x,y)P(x_{k}⋄x_{l}|x)P(y_{m}⋄y_{n}|y). \end{array}$$

*P*(*x*_*k *_◇ *x*_*l*_|***x***) and *P*(*y*_*m *_◇ *y*_*n*_|***y***) are the base-pairing probabilities that the particular positions *x*_*k *_and *x*_*l*_, *y*_*m *_and *y*_*n*_, respectively, form base pairs; these probabilities are computed by McCaskill's algorithm [27].

$\tilde{P}$(*x*_*k *_~ *y*_*m*_|***x***, ***y***) and $\tilde{P}$(*x*_*l *_~ *y*_*n*_|***x***, ***y***) are the posterior probabilities modified by probability consistency transformation [32], which is computed as follows.

(3) $$\tilde{P}(x_{k}\sim y_{m}|x,y)=\frac{1}{\left|S\right|}\sum_{z\in S}\sum_{r\in \{1,...,\left|z\right|\}}P(x_{k}\sim z_{r}|x,z)P(z_{r}\sim y_{m}|z,y),$$

where *S *is the set of RNA sequences to be aligned. In this transformation, the probability of specific nucleotides of two sequences being aligned are replaced by the average over the products of probabilities that the two nucleotides are aligned to the same nucleotides in arbitrary third sequences. This calculation requires *O*(*N*^3^*L*^3^) in time and *O*(*N*^2^*L*^2^) in memory. The probability consistency transformations are applied twice in current implementation.

*P*(*x*_*k *_~ *z*_*r*_|***x***, ***z***) is the posterior probability, i.e. the sum of the probabilities that particular positions of the two sequences, *x*_*k *_and *z*_*r*_, are aligned in some alignment; this is computed by the forward-backward algorithm of the pair Hidden Markov Model (pair HMM) [31] for pairwise alignment of the sequences. Our new matching scores in (1) are defined as follows.

(4) $$s(i,j)=\sum_{0\leq w<W}\hat{P}(x_{p(X_{i})+w}\sim y_{p(Y_{j})+w},x_{p({X^{\prime }}_{i})+W-1-w}\sim y_{p({Y^{\prime }}_{j})+W-1-w})$$

(5) $$\delta_{s}(i,j)=\hat{P}(x_{p(X_{i})+W-1}\sim y_{p(Y_{j})+W-1},x_{p({X^{\prime }}_{i})}\sim y_{p({Y^{\prime }}_{j})})$$

where ${X^{\prime }}_{i}/{Y^{\prime }}_{j}$ are the complementary stem components of *X*_*i*_/*Y*_*j*_.

The sum of the probabilities, not the logarithms of the probabilities, is used for the matching score, in an effort to maximize the number of correctly aligned bases including the implicit prediction of the base pairs (MEA principle).

#### Alignment of loop region

The remaining loop regions (except the selected common stems) are aligned by using the consistency-transferred posterior probabilities, $\tilde{P}$(*x*_*k *_~ *y*_*m*_|***x***, ***y***), as the matching scores. The probabilities, not the logarithms of the probabilities, again are used, according to the MEA principle. The recursion is shown following.

$$A(k,m)=\max\{\begin{array}{l} A(k-1,m-1)+\tilde{P}(x_{k}\sim y_{m}|x,y)\\ A(k-1,m)\\ A(k,m-1) \end{array}$$

**Figure 3:**
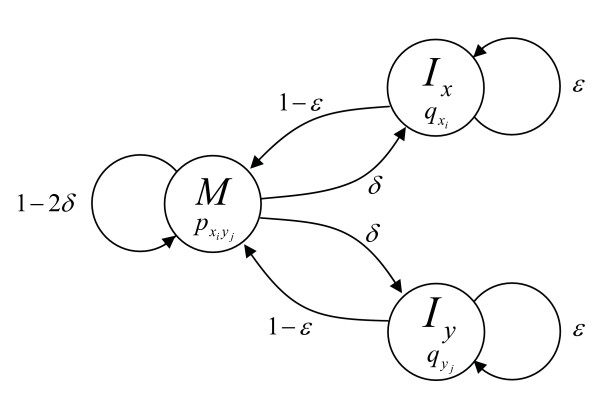
**A pair-HMM for pairwise sequence alignment**. A pair-HMM is used for alignment of loop regions and calculation of the posterior probabilities in score function. The state *M *has emission probability distribution $p_{x_{i}y_{j}}$ for emitting an aligned pair *x*_*i *_and *y*_*j*_. The state *I*_*x *_has distributions $q_{x_{i}}$ for emitting symbol *x*_*i *_against a gap. The state *I*_*y *_has distributions $q_{y_{j}}$ for emitting symbol *y*_*j *_against a gap. The parameters *δ *and *ε *are the state transition probabilities.

Emission and transition probabilities for the pair HMM in MXSCARNA (Figure 3) were trained via Expectation-Maximization (EM) on a set of unaligned sequences that is extracted from the Rfam database and that do not overlap the sequences of the dataset for subsequent experiments.

### Computational Experiments

#### Datasets

To test the empirical performance of MXSCARNA, we used three datasets for the benchmark multiple alignments: an original multiple alignment dataset, the BRAlibaseII multiple alignment dataset [33], and Kiryu et al.'s multiple alignment dataset [21].

Our original dataset comprised 1669 multiple alignments of 5 sequences, the secondary structures of which have been published, obtained from the Rfam 7.0 database [34]. There are 27 families of RNA sequences in the dataset and the sequence identities varied from 35% to 100%. Sequences that included bases other than A, C, G, and U were removed because some of the alignment programs were unable to align them. The BRAlibaseII benchmark dataset included 481 multiple alignments of 5 sequences. The sequences of each multiple alignment were extracted from tRNA, Intron_gpII, 5S_rRNA, and U5 families in the Rfam 5.0 database and the signal recognition particle RNA family (SRP) in the SRPDB database [35]. Because the dataset did not include consensus secondary structure annotations to the alignments, we used the secondary structure annotations recovered by Kiryu et al. [21].

Kiryu et al.'s multiple alignment benchmark dataset was generated from selected seed alignments in the Rfam 7.0 database that have published consensus structures [21]. For each sequence family, as many as 1000 random combinations of 10 sequences were generated. The alignments whose mean pairwise sequence identity exceeded 95% and whose gap characters accounted for more than 30% of the total number of characters aligned were removed. As such, this dataset consisted of 85 multiple alignments of 10 sequences, generated from 17 sequence families, with five alignments for each. The dataset was reasonably divergent, and its mean length varied from 54 to 291 bases, and mean pairwise sequence identities varied from 40% to 94%.

#### Evaluation measures

The qualities of the alignments were evaluated by the Sum-of-Pairs Score (SPS) for the accuracy of the alignments and by the Matthews Correlation Coefficient (MCC) [36] for the accuracy of the secondary structure predictions. The SPS and MCC of the alignment to be evaluated (named as a *test alignment*) for the reference alignment were defined as follows. The SPS was defined as the proportion of correctly aligned nucleotide pairs:

$$\text{SPS}=\frac{\sum_{i=1}^{I}{\text{SP}}_{i}^{t}}{\sum_{j=1}^{J}{\text{SP}}_{j}^{r}}$$

where *I *is the number of columns in the test alignment, *J *is the number of columns in the reference alignment, on column *i *in the test alignment ${\text{SP}}_{i}^{t}$ is the total number of "correct" nucleotide pairs which also appear in the reference alignment, on column *j *in the reference alignment ${\text{SP}}_{j}^{r}$ is the total number of nucleotide pairs. The MCC was defined as

$$\begin{array}{lll} \text{MCC} & = & \frac{\text{TP}\times \text{TN}-\text{FP'}\times \text{FN}}{\sqrt{\left(\text{TP}+\text{FP'})(\text{TP}+\text{FN})(\text{TN}+\text{FP'})(\text{TN}+\text{FN}\right)}},\\ \text{FP'} & = & \text{FP}-\xi , \end{array}$$

where TP indicates the number of correctly predicted base pairs, TN the number of base pairs that were correctly predicted as unpaired, FP the number of incorrectly predicted base pairs, and FN the number of true base pairs that were not predicted. The term *ξ *accounts for predicted base pairs that were not present in the reference structure but were compatible with it. Compatible base pairs are not true positives but have to be neither inconsistent (one or both nucleotides being a part of a different base pair in the reference structure) nor pseudo-knotted with respect to the reference structure [37]. In order to calculate MCC for each test alignment, the reference alignment and the "correct" consensus secondary structure are taken from the database. In order to compare the accuracies of the alignments in terms of the implicitly predicted common secondary structures, the common secondary structures for each test alignment by the alignment programs were predicted by the Pfold program [5].

#### Comparison of accuracies with those of other aligners

To compare the accuracies of the alignment methods we used a Linux machine with an AMD Opteron processor (2 GHz and 4 GB RAM).

We compared the performance of MXSCARNA with that of Murlet [21], ProbCons [32], MAFFT [38], ClustalW [7], StrAl [18], MARNA [17], RNASampler [19], RNAlara [20], FoldalignM [15], Locarna [16], PMmulti [12], and Stemloc [13] on the three datasets described earlier. Whereas ProbCons, MAFFT, and ClustalW align RNA sequences on the basis of sequence similarities only, StrAl, MARNA, RNASampler, RNAlara, FoldalignM, Locarna, PMmulti, Stemloc, and Murlet weigh both sequence similarities and secondary structures. The command line options for the programs in the experiments are shown in Table 1. The results for the original dataset are shown in Table 2. Because MARNA, Locarna, FoldalignM, PMmulti, and Stemloc impose high time and memory demands, those programs were executed only on families of which the average sequence lengths were less than or equal to 100 bases. The SPS of MXSCARNA was comparable to those of Murlet and ProbCons, which currently are the best performing aligners [21]. In addition, the MCC of MXSCARNA was one of the highest among aligners. In particular, the MCC of MXSCARNA is similar to that of Stemloc, which aligns only short sequences that have simple secondary structures.

**Table 1:** Command line options for the programs in the experiments. This table summarizes multiple alignment programs and their command line options used in the paper.

Program	Command
MXSCARNA	./mxscarna <input_filename>
Murlet	./murlet -max_time = 100 <input_filename>
ProbCons	./probcons <input_filename>
MAFFT	./mafft <input_filename>
ClustalW	./clustalw <input_filename>
StrAl	./stral <input_filename>
RNASampler	perl RNASampler_driver.pl -p <input_dir> -q <input_filename> -i 15 -S 100
RNAlara	./lara -i <input_filename>
Locarna	./mlocarna -struct-local = false -sequ-local = false <input_filename>
FoldalignM-Foldalign	perl FoldalignM_Foldalign.pl -f <input_filename>
FoldalignM-McCaskill	java FoldalignM_McCaskill <input_filename>
MARNA	perl marna.pl -g 2 <input_filename>
PMmulti	perl pmmulti.pl <input_filename>
stemloc	./stemloc -g -m -slow <input_filename>

**Table 2:** Accuracies for the original multiple alignment dataset. SPS and MCC values (%) for the original multiple alignment dataset are presented. Each family has 5 RNA sequences. Family: Rfam family name. %id: average sequence identity. length: average sequence length (bases) in each family. Average(all): the average SPS or MCC for all families. Average(sub): the average SPS or MCC for the subset of families with an average sequence length of less than or equal to 100 bases. Because PMmulti and Stemloc were unable to align all data, the proportion of data that was aligned is given in parentheses as no. of sequences aligned/total no. of sequences. FoldalignM consists of two modes: FoldalgnM_FoldalignM and FoldalignM_McCaskill, which are separately evaluated and indicated as FoldalignM(1) and FoldalignM(2) respectively.

SPS:Family	%id	length	MXSCARNA	Murlet	ProbCons	MAFFT	ClustalW	StrAl	RNASampler
IRE	62	29	98	96	93	89	77	83	92
s2m	78	43	96	96	96	96	96	96	95
UnaL2	78	54	95	98	98	96	92	94	82
Hammerhead_3	71	55	96	94	93	89	83	89	91
SECIS	42	64	65	66	65	52	45	56	46
sno_14q_I_II	71	74	90	95	96	93	93	87	71
tRNA	48	76	87	87	87	84	76	82	87
ctRNA_pGA1	74	80	88	86	85	88	84	86	89
Tymo_tRNA	70	83	84	83	82	75	75	79	77
Y	64	95	71	72	72	73	65	64	65
SRP_bact	52	95	71	71	70	66	70	62	66
Purine	55	100	74	77	77	78	75	81	69
5S_rRNA	60	117	88	89	89	86	86	86	83
S_box	66	130	79	80	78	78	68	72	67
U4	67	141	79	81	80	79	79	78	69
RFN	65	150	86	87	87	88	81	80	81
5_8S_rRNA	67	154	91	93	93	90	88	89	78
U1	60	158	81	81	79	79	79	76	74
Telomerase_cil	56	171	48	50	49	43	38	41	37
Lysine	50	180	78	81	79	72	70	76	72
U2	66	185	76	76	75	71	71	73	71
U17	75	214	91	93	93	89	89	87	81
U3	51	246	43	44	44	47	41	40	34
SRP_euk_arch	46	294	50	56	47	42	42	48	44
tmRNA	46	373	48	50	50	47	46	39	42
RnaseP_bact_b	64	387	82	80	79	78	74	74	66
Telomerase_vert	66	463	69	70	69	69	66	64	65

Average(all)			78	79	78	75	72	73	70

Average(sub)			85	85	85	82	78	80	78

SPS:Family	RNAlara	Locarna	FoldalignM(1)	FoldalignM(2)	MARNA	PMmulti	Stemloc		

IRE	85	88 (100/100)	87 (99/101)	85 (97/100)	92	90 (101/101)	87 (101/101)		
s2m	96	99 (43/50)	95 (50/50)	95 (47/50)	94	98 (44/50)	93 (50/50)		
UnaL2	93	93 (78/89)	89 (75/89)	86 (87/89)	88	87 (75/89)	75 (89/89)		
Hammerhead_3	88	80 (100/100)	79 (99/100)	77 (97/100)	89	87 (77/100)	94 (100/100)		
SECIS	48	47 (62/63)	46 (63/63)	44 (63/63)	49	39 (63/63)	60 (60/63)		
sno_14q_I_II	78	76 (98/98)	73 (98/98)	61 (97/98)	67	73 (97/98)	89 (97/98)		
tRNA	91	82 (103/103)	90 (100/103)	78 (97/103)	59	86 (103/103)	88 (103/103)		
ctRNA_pGA1	86	74 (20/28)	75 (28/28)	74 (27/28)	79	72 (20/28)	84 (28/28)		
Tymo_tRNA	79	78 (49/59)	68 (59/59)	68 (56/59)	66	71 (49/59)	57 (57/59)		
Y	56	49 (21/24)	50 (24/24)	47 (24/24)	60	43 (11/24)	68 (23/24)		
SRP_bact	63	62 (70/70)	64 (70/70)	56 (67/70)	56	61 (63/70)	60 (67/70)		
Purine	64	65 (45/45)	64 (45/45)	62 (45/45)	64	65 (45/45)	37 (45/45)		
5S_rRNA	85								
S_box	57								
U4	71								
RFN	78								
5_8S_rRNA	81								
U1	74								
Telomerase_cil	32								
Lysine	59								
U2	71								
U17	79								
U3	32								
SRP_euk_arch	42								
tmRNA	34								
RnaseP_bact_b	66								
Telomerase_vert	51								

Average(all)	68								

Average(sub)	77	74	73	69	72	73	74		

MCC:Family	%id	length	MXSCARNA	Murlet	ProbCons	MAFFT	ClustalW	StrAl	RNASampler

IRE	62	29	90	91	75	72	65	43	89
S2m	78	43	84	83	84	84	84	84	81
UnaL2	78	54	52	70	51	53	51	50	51
Hammerhead_3	71	55	99	95	93	86	72	79	96
SECIS	42	64	76	60	55	35	23	37	73
sno_14q_I_II	71	74	93	98	93	93	91	91	95
tRNA	48	76	91	89	86	84	76	83	95
ctRNA_pGA1	74	80	96	93	88	89	81	92	94
Tymo_tRNA	70	83	87	85	75	73	72	80	90
Y	64	95	95	85	86	83	67	83	94
SRP_bact	52	95	81	72	54	50	58	57	83
Purine	55	100	90	94	90	86	80	84	91
5S_rRNA	60	117	75	79	69	70	69	70	70
S_box	66	130	90	87	86	81	76	81	86
U4	67	141	75	71	62	62	54	65	67
RFN	65	150	84	83	84	84	82	83	82
5_8S_rRNA	67	154	58	51	47	45	41	46	52
U1	60	158	70	68	61	56	60	57	71
Telomerase	56	171	65	41	28	21	24	31	60
Lysine	50	180	87	90	76	66	63	71	89
U2	66	185	73	76	58	51	62	59	77
U17	75	214	79	80	78	76	75	72	72
U3	51	246	46	26	22	19	46	21	39
SRP_euk_arch	46	294	72	75	46	37	35	49	72
tmRNA	46	373	51	54	50	49	43	42	49
RNaseP_bact_b	64	387	73	58	63	58	53	60	37
Telomerase	66	463	64	51	47	44	40	36	53

Average(all)			78	74	67	63	61	63	74

Average(sub)			86	85	77	74	68	72	86

MCC:Family	RNAlara	Locarna	FoldalignM(1)	FoldalignM(2)	MARNA	PMmulti	Stemloc		

IRE	81	89	85	89	82	89	81		
s2m	84	83	85	86	79	85	84		
UnaL2	53	53	53	51	51	50	45		
Hammerhead_3	95	94	87	91	95	91	98		
SECIS	63	74	74	75	57	67	78		
sno_14q_I_II	92	87	92	84	81	93	85		
tRNA	95	87	95	87	67	90	95		
ctRNA_pGA1	97	95	96	96	95	94	95		
Tymo_tRNA	85	75	87	82	62	82	83		
Y	86	87	94	89	87	83	93		
SRP_bact	65	87	86	83	63	80	69		
Purine	77	89	88	88	82	86	89		
5S_rRNA	72								
S_box	72								
U4	60								
RFN	79								
5_8S_rRNA	46								
U1	60								
Telomerase	39								
Lysine	58								
U2	63								
U17	63								
U3	21								
SRP_euk_arch	42								
tmRNA	40								
RNaseP_bact_b	65								
Telomerase	28								

Average(all)	65								

Average(sub)	81	83	85	83	75	83	83		

The results from the BRAlibaseII benchmark multiple alignment dataset are shown in Table 3. Because of their prohibitive requirements for memory and time, Stemloc, FoldalignM, PMmulti, and MARNA were not applied to the SRP family data. Again, MXSCARNA was comparable to Murlet and ProbCons in terms of SPS and one of the best performers among multiple aligners according to the MCC. These trends continue in Table 4, which contains the results from Kiryu et al.'s benchmark dataset comprising 10 sequences for each alignment.

**Table 3:** Accuracies for the BRAlibaseII multiple alignment dataset. SPS and MCC values (%) for the BRAlibaseII multiple alignment dataset are presented. Each family has 5 RNA sequences. Family: Rfam family name. %id: average sequence identity. length: average sequence length (bases) in each family. Average(all): the results of the average value of the SPS or MCC for all families. Average(sub): the average SPS or MCC for the subset of families with an average sequence length of less than or equal to 100 bases. Because PMmulti and Stemloc were unable to align all data, the proportion of data that was aligned is given in parentheses as no. of sequences aligned/total no. of sequences. FoldalignM consists of two modes: FoldalgnM_FoldalignM and FoldalignM_McCaskill, which are separately evaluated and indicated as FoldalignM(1) and FoldalignM(2) respectively.

SPS:Family	%id	length	MXSCARNA	Murlet	ProbCons	MAFFT	ClustalW	StrAl	RNASampler	
tRNA	69	76	91	91	91	89	85	89	92	
Intron_gpII	64	80	79	80	80	77	75	79	74	
5S_rRNA	70	117	89	90	90	89	88	89	90	
U5	72	118	74	75	76	72	72	73	78	
SRP	67	300	88	88	88	87	87	86	82	

Average(all)			84	85	85	83	81	83	83	

Average(sub)			83	84	84	82	80	82	83	

SPS:Family	RNAlara	Locarna	FoldalignM(1)	FoldalignM(2)	MARNA	PMmulti	Stemloc			

tRNA	95	93 (98/98)	94 (97/98)	90 (91/98)	79 (98/98)	90 (89/98)	88 (98/98)			
Intron_gpII	75	71 (89/92)	70 (92/92)	67 (89/92)	76 (92/92)	77 (61/92)	77 (92/92)			
5S_rRNA	93	92 (89/89)	92 (88/89)	89 (89/89)	58 (78/89)	85 (89/89)	72 (89/89)			
U5	80	77 (109/109)	72 (108/109)	69 (107/109)	85 (74/109)	56 (105/109)	64 (109/109)			
SRP	82	83 (84/93)								

Average(all)	85	83								

Average(sub)	86	83	82	79	74	77	75			

MCC:Family	%id	length	MXSCARNA	Murlet	ProbCons	MAFFT	ClustalW	StrAl	RNASampler	RNAlara

tRNA	69	76	94	92	91	90	83	88	94	93
Intron_gpII	64	80	82	80	77	76	74	74	80	79
5S_rRNA	70	117	71	69	67	68	67	69	69	70
U5	72	118	80	75	70	66	66	69	77	72
SRP	67	300	75	72	68	67	68	65	71	63

Average(all)			80	78	75	73	72	73	78	76

Average(sub)			82	79	76	75	72	75	80	79

MCC:Family	Locarna	FoldalignM(1)	FoldalignM(2)	MARNA	PMmulti	Stemloc				

tRNA	92	96	92	80	93	93				
Intron_gpII	80	76	80	78	76	76				
5S_rRNA	71	72	71	59	70	68				
U5	74	70	69	60	61	78				
SRP	73									

Average(all)	78									

Average(sub)	79	79	78	69	75	79				

**Table 4:** Accuracies for Kiryu et al.'s dataset. SPS and MCC values (%) for Kiryu et al.'s dataset are presented. Each family has 10 RNA sequences. Family: Rfam family name. %id: average sequence identity. length: average sequence length (bases) in each family. Average(all): the results of the average value of the SPS or MCC for all families. Average(sub): the average SPS or MCC for the subset of families with an average sequence length of less than or equal to 100 bases. Because PMmulti and Stemloc were unable to align all data, the proportion of data that was aligned is given in parentheses as no. of sequences aligned/total no. of sequences. FoldalignM consists of two modes: FoldalgnM_FoldalignM and FoldalignM McCaskill, which are separately evaluated and indicated as FoldalignM(1) and FoldalignM(2) respectively.

SPS:Family	%id	length(nt)	MXSCARNA	Murlet	ProbCons	MAFFT	ClustalW	StrAl	RNAlara
UnaL2	73	54	92	95	95	91	84	86	87
SECIS	41	64	70	73	68	44	35	59	53
tRNA	45	73	87	90	87	76	62	75	91
sno_14q_I_II	64	75	82	92	92	91	80	75	72
SRP_bact	47	93	58	61	61	60	61	48	56
THI	55	105	77	83	82	78	58	65	65
S_box	66	107	86	88	88	82	82	77	77
5S_rRNA	57	116	84	85	85	81	82	79	83
Retroviral_psi	92	117	97	97	97	97	96	97	97
RFN	66	140	89	91	90	91	83	80	86
5_8S_rRNA	61	154	85	88	87	84	78	81	75
U1	59	157	74	77	75	73	71	66	66
Lysine	49	181	75	77	75	66	60	68	59
U2	62	182	71	74	73	68	65	67	69
T-box	45	244	44	50	50	43	34	32	15
IRES_HCV	94	261	96	96	96	96	96	83	96
SRP_euk_arch	40	291	42	42	40	36	34	40	39

Average(all)			77	80	79	74	68	69	70

Average(sub)			82	86	85	80	73	75	77

SPS:Family	RNASampler	Locarna	FoldalignM(1)	FoldalignM(2)	MARNA	PMmulti	Stemloc		

UnaL2	72	88	68	60	83 (5/5)	69 (5/5)	82 (5/5)		
SECIS	54	49	42	41	47 (5/5)	35 (5/5)	82 (5/5)		
tRNA	82	79	84	66	54 (5/5)	69 (5/5)	91 (5/5)		
sno_14q_I_II	64	57	45	34	49 (5/5)	39 (3/5)	77 (5/5)		
SRP_bact	54	52	55	51	43 (5/5)	36 (4/5)	47 (3/5)		
THI	68	65	65	62	62 (4/5)	58 (5/5)	71 (5/5)		
S_box	76	63	57	57	78 (5/5)	44 (5/5)	84 (5/5)		
5S_rRNA	77	79	74	70	71 (5/5)	57 (5/5)	77 (3/5)		
Retroviral_psi	96	95	91	91	96 (5/5)	87 (5/5)	75 (5/5)		
RFN	82	72	73	63	77 (5/5)	58 (4/5)	80 (5/5)		
5_8S_rRNA	69	75	56	31	64 (5/5)	58 (5/5)	73 (1/5)		
U1	63	68			50 (5/5)				
Lysine	71	55			58 (5/5)				
U2	65	64			65 (1/5)				
T-box	32	15			22 (5/5)				
IRES_HCV	93	75			92 (3/5)				
SRP_euk_arch	33	40			37 (5/5)				

Average(all)	68	64			62				

Average(sub)	72	70	64	57	52	60	76		

MCC:Family	%id	length(nt)	MXSCARNA	Murlet	ProbCons	MAFFT	ClustalW	StrAl	RNAlara

UnaL2	73	54	42	41	46	36	24	32	44
SECIS	41	64	78	78	59	20	23	45	70
tRNA	45	73	93	97	91	85	65	85	97
sno_14q_I_II	64	75	87	91	91	91	66	75	87
SRP_bact	47	93	66	56	46	49	54	52	69
THI	55	105	71	70	70	62	38	48	58
S_box	66	107	90	89	87	79	77	75	76
5S_rRNA	57	116	75	67	62	64	53	66	69
Retroviral_psi	92	117	86	86	86	84	86	86	86
RFN	66	140	67	71	72	73	71	60	70
5_8S_rRNA	61	154	38	43	35	16	14	26	33
U1	59	157	69	61	57	56	61	52	56
Lysine	49	181	83	81	71	33	52	61	64
U2	62	182	74	71	56	38	39	58	68
T-box	45	244	72	78	80	51	41	26	0
IRES_HCV	94	261	63	62	62	63	26	34	63
SRP_euk_arch	40	291	70	63	40	21	23	38	33

Average(all)			72	71	65	54	48	54	61

Average(sub)			72	72	68	60	52	59	69

MCC:Family	RNASampler	Locarna	FoldalignM(1)	FoldalignM(2)	MARNA	PMmulti	Stemloc		

UnaL2	51	42	57	50	36	18	39		
SECIS	78	80	75	80	40	64	77		
tRNA	94	96	95	89	51	91	98		
sno_14q_I_II	84	95	95	86	79	77	87		
SRP_bact	73	74	80	75	47	44	64		
THI	72	60	50	51	59	41	77		
S_box	86	82	83	87	83	55	88		
5S_rRNA	62	75	72	73	59	58	66		
Retroviral_psi	86	88	76	89	84	73	87		
RFN	69	69	67	70	65	58	72		
5_8S_rRNA	38	40	43	41	21	14	34		
U1	64	73			45				
Lysine	86	80			66				
U2	79	65			66				
T-box	44	1			2				
IRES_HCV	63	47			61				
SRP_euk_arch	62	64			52				

Average(all)	70	66			54				

Average(sub)	72	73	72	72	60	54	72		

All results are summarized in Table 5.

**Table 5:** Summary of accuracies for all three datasets. The summary of SPS and MCC values (%) for all three multiple alignment datasets are presented. Average(all): the results of the average value of the SPS or MCC for all families. Average(sub): the average SPS or MCC for the subset of families. FoldalignM consists of two modes: FoldalgnM_FoldalignM and FoldalignM_McCaskill, which are separately evaluated and indicated as FoldalignM(1) and FoldalignM(2) respectively.

Dataset		MXSCARNA	Murlet	ProbCons	MAFFT	ClustalW	StrAl	RNASampler
original dataset	Average(all)	78/**78**	**79**/74	78/67	75/63	72/61	73/63	70/74
	Average(sub)	**85**/**86**	**85**/85	**85**/77	82/74	78/68	80/72	78/**86**

BRAlibaseII	Average(all)	84/**80**	**85**/78	**85**/75	83/73	81/72	83/73	83/78
	Average(sub)	83/**82**	84/79	84/76	82/75	80/72	82/75	83/80

Kiryu et al.'s dataset	Average(all)	77/**72**	**80**/71	79/65	74/54	68/48	69/54	68/70
	Average(sub)	82/72	**86**/72	85/68	80/60	73/52	75/59	72/72

		RNAlara	Locarna	FoldalignM(1)	FoldalignM(2)	MARNA	PMmulti	Stemloc

original dataset	Average(all)	68/65						
	Average(sub)	77/81	74/83	73/85	69/83	72/75	73/83	74/83

BRAlibaseII	Average(all)	**85**/76	83/78					
	Average(sub)	**86**/79	83/79	82/79	79/78	74/69	77/75	75/79

Kiryu et al.'s dataset	Average(all)	70/61	64/66			62/54		
	Average(sub)	77/69	70/**73**	64/72	57/72	60/60	52/54	76/72

#### Evaluation of new score function

In order to evaluate the performance of our new score function (4), we compared it in pairwise alignment with the previous score function of SCARNA, which is a linear combination of RIBOSUM score, stacking energy, loop-distance penalty, base-pairing probability. Dowell's dataset [39], which consists of R100 dataset and percid dataset, are used for the evaluation. R100 is a dataset which consists of 100 pairwise alignments chosen randomly from tRNA and 5SrRNA families in Rfam 7.0 database [34] and percid is a balanced dataset of 100 pairwise alignments from the same families.

The SPS and MCC are shown in Table 6. It is observed that the new score function of MXSCARNA outperformed the previous score function of SCARNA.

**Table 6:** Accuracy of new score function. The comparison of new score function of MXSCARNA and the old one which was used in SCARNA in terms of pairwise alignment. The SPS and MCC values (%) are used as accuracy measure for alignments. R100 is a dataset which consists of 100 pairwise alignments chosen randomly from tRNA and 5SrRNA families in Rfam 7.0 database [34] and percid is a sequence identitly balanced dataset which also consists of 100 pairwise alignments from these families.

dataset	score function	SPS	MCC
R100	MXSCARNA	**90**	**77**
	SCARNA	84	74
percid	MXSCARNA	**79**	**71**
	SCARNA	78	69

#### Time and memory

The computational complexities of the proposed method for *N *sequences of length *L *were evaluated as follows. The construction of the guide tree using the alignments of all pairs of the sequences required *O*(*N*^2^*L*^2^) in time and *O*(*L*^2 ^+ *N*^2^) in memory. The calculation of base-pairing probability matrices for *N *sequences by McCaskill's algorithm [27] required *O*(*NL*^3^) in time and *O*(*NL*^2^) in memory. The probability consistency transformation (see (3) in Method) required *O*(*N*^3^*L*^3^) in time and *O*(*N*^2^*L*^2^) in memory. Pairwise alignment of stem component sequences required *O*(*N*^2^*L*^2^) in time and memory as is explained in Method. Therefore, the total computational complexities were *O*(*N*^3^*L*^3^) in time and *O*(*N*^2^*L*^2^) in memory. For the base-pairing probabilities, the computational time for each sequence can be reduced to *O*(*LW*^2^) by restricting the maximum distance of the base pairs to a fixed constant *W *[40]. The computation of probability consistency transformation for a pair of sequences can also be calculated in *O*(*L*^2^) time by restricting the effective width of transformation to a fixed value. Those reductions reduce total time complexity to *O*(*N*^3^*L*^2^). We will address those improvements in future work.

**Figure 4:**
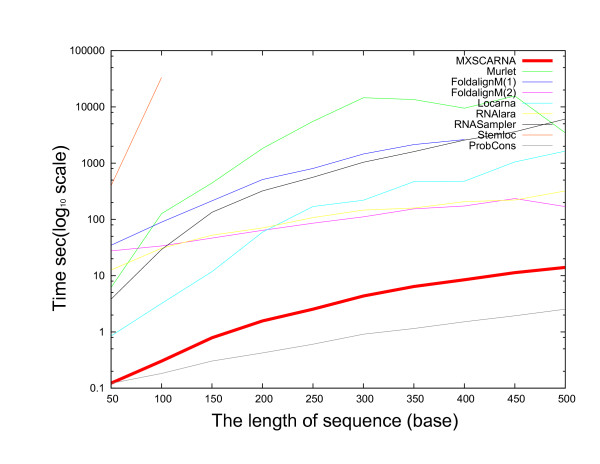
**Comparison of multiple alignment tools in execution time for various lengths of the sequences (maximal sequence length, 500 bases)**. The relationships between the length of the sequences (maximum, 500 bases) and the execution time for several multiple alignment tools are plotted. A set of randomly generated sequences of the same length is used for each alignment.

**Figure 5:**
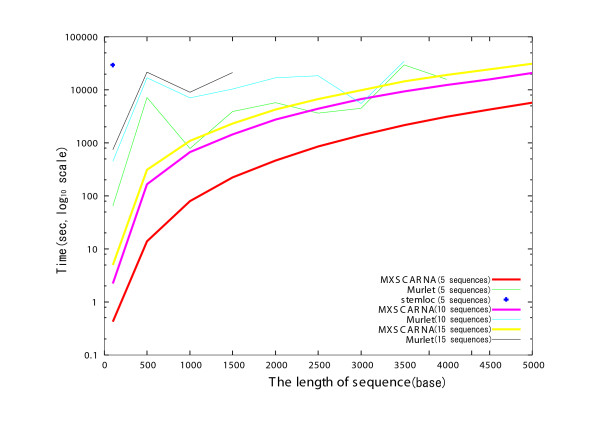
**Comparison of multiple alignment tools in execution time for various lengths of the sequences (maximal sequence length, 5000 bases)**. The relationships between the length of the sequences (maximum, 5000 bases) and the execution time for MXSCARNA, Murlet and Stemloc are plotted. A set of randomly generated sequences of a same length is used for each alignment. The number of the sequences used for the alignment is indicated after the names of the tools. The accuracies for the sequences longer than 500 bases have not been evaluated.

**Figure 6:**
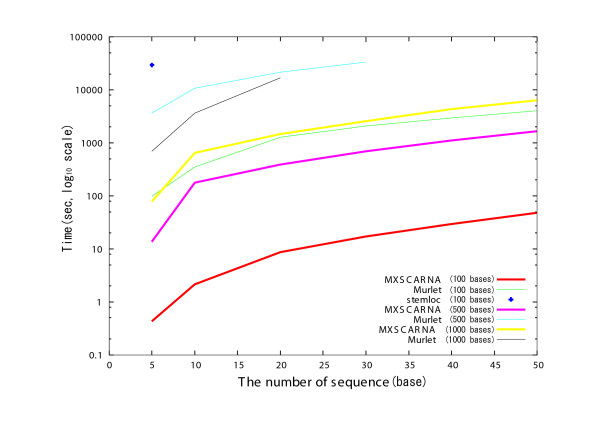
**Comparison of various multiple alignment tools in execution time and the number of sequences**. The relationships between the number of the sequences and the execution time for MXSCARNA, Murlet and Stemloc are plotted. A set of randomly generated sequences of a same length is used for each alignment. The lengths of the sequences used for the alignment is indicated after the names of the tools.

Comparisons of alignment tools in regard to execution time for nucleotide sequences of various lengths are presented in Figures 4 and 5. Randomly generated sequences were allocated into groups of the same lengths and were used for alignment. Stemloc aligned sequences of not more than 100 bases; FoldalignM and Locarna were faster than Stemloc and aligned sequences of 500 bases or less. Because the lengths of the sequences were the same in each alignment task, the banded Dynamic Programming (DP) technique of these methods was effective. Although the Murlet program returned results for sequences as long as 4000 bases in the best case, it was much slower than MXSCARNA. MXSCARNA required only 17 seconds to align 5 sequences of 500 bases and returns alignments for sequences as long as 5000 bases, though the accuracies for sequences longer than 500 bases have not yet been evaluated. Similar comparisons for various numbers of the sequences are presented in Figure 6. The execution time of MXSCARNA is acceptable even for 50 sequences.

#### Sequence identities and alignment accuracies

**Figure 7:**
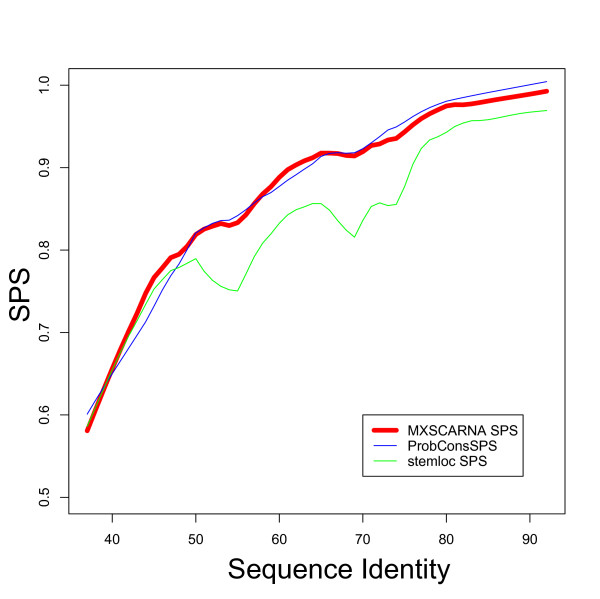
**Relationship between sequence similarities and SPS**. The relationship between sequence similarities and accuracies according to sum-of-pairs score (SPS) is shown. Lines are smoothed by local weighted regression.

**Figure 8:**
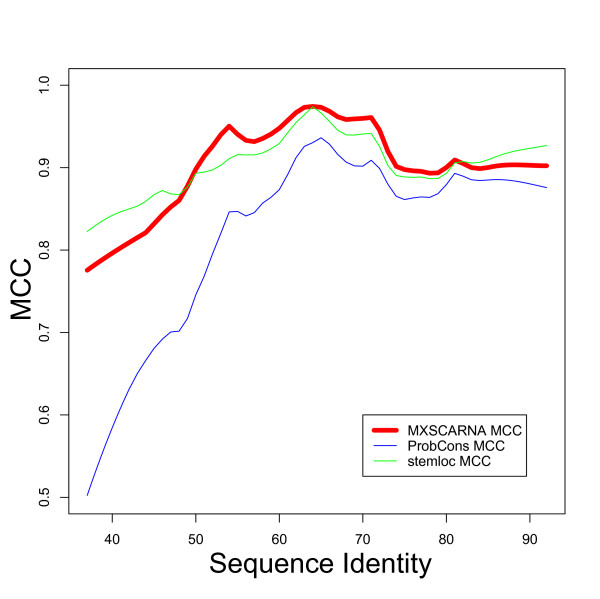
**Relationship between sequence similarities and MCC**. The relationship between sequence similarities and accuracies according to the Matthews correlation coefficient (MCC) is shown. Lines are smoothed by local weighted regression.

Alignment methods based only on sequence similarities often fail to capture common secondary structures among their alignments, especially when the similarities between sequences are low. In contrast, current alignment methods that rely on information about secondary structures tend to produce inaccurate alignments for sequences of moderate to high similarity by putting too much weight on common secondary structures. The relationships between accuracy and sequence identity for three alignment tools MXSCARNA, ProbCons, and Stemloc are shown in Figures 7 and 8. ProbCons, one of the best of the aligners that ignore information regarding secondary structure, maintains a high SPS throughout low to high sequence similarities, but MCC markedly drops for low sequence identities. Stemloc, one of the best structural aligners (as seen in the previous section), achieved robust accuracies in MCC but failed to compete among the other aligners in regard to SPS for moderate sequence identities. MXSCARNA, which incorporates information on Maximum Expected Accuracy (MEA) alignment in its structural alignments, yielded robust accuracies in terms of both SPS and MCC throughout the tested range of sequence similarities.

## Availability and requirements

Project name: ncRNA.org project;

Project home page: ;

Operating systems: Linux with gcc 3.0 and Cygwin with gcc 3.4;

Programming language: C++;

License: free software, except for inclusion to comertical software;

The source code of MXSCARNA and its web server, the dataset and its references are available at . On the web server *W *and *τ *correspond to "SCSLENGTH" and "BASEPROBTHRESHHOLD" respectively, and "BASEPAIRSCORECONST" is a parameter of McCaskill-MEA [6] used for the secondary structure prediction, which controls the sensitivity and the specificity of the prediction (*α *in equation 4 in [6]).

## Conclusion

We have developed MXSCARNA, a new structural multiple aligner of RNA sequences, which progressively applies the pairwise alignment algorithm used in SCARNA. The accuracies of MXSCARNA in terms of SPS and MCC were evaluated for three datasets: an original dataset, the BRAlibaseII benchmark multiple alignment dataset, and Kiryu et al.'s multiple alignment dataset. MXSCARNA's accuracies were at least comparable to those of current state-of-art aligners. In addition, the accuracies of MXSCARNA were robust over a broad range of sequence similarities, whereas the other aligners tested showed reductions in SPS or MCC. The computational complexities of MXSCARNA were evaluated as *O*(*N*^3^*L*^3^) in time and *O*(*N*^2^*L*^2^) in memory for *N *sequences of length *L*. In the comparison of execution time for benchmark datasets, MXSCARNA was by far the fastest among the structural aligners and was fast enough for large-scale analyses. MXSCARNA aligns even 5000-base RNA sequences with acceptable computational costs though the accuracies of alignments for long sequences are not yet confirmed. The source code of MXSCARNA and its web server are available at the web site [41].

## Authors' contributions

YT and KA developed the algorithm, and together they wrote the manuscript with the help of TK. YT implemented the algorithm into the software (MXSCARNA) and executed all of the computational experiments. HK contributed to the design of the new score function and closely collaborated in the computational experiments. TK helped to design the computational experiments and to write the manuscript. With the help of YT, KA organized the development of the web server. All the authors have read and approved the final version of the manuscript.
